# Researching and designing educational games on the basis of “self-regulated learning theory”

**DOI:** 10.3389/fpsyg.2022.996403

**Published:** 2022-11-11

**Authors:** Feng Jiang, Dayan Shangguan

**Affiliations:** School of Art and Design, Beijing Forestry University, Beijing, China

**Keywords:** educational games, learning motivation, motivation regulation, framework design, game design

## Abstract

As one of the important research fields of educational technology, the potential of educational games has been widely recognized by academic researchers. However, in terms of practical application, it is difficult to balance education and recreation, and problems have also arisen in learners’ cognitive development and skill enhancement. On this basis, this paper initially compares the educational and entertainment aspects of educational games from a learning motivation perspective. It draws on the theory of self-regulated learning and ARCS learning to establish an ARCS learning motivation model and educational game design framework. Finally, it develops a bio evolution education game that is based on this framework, and this verifies that this framework can feasibly guide practice. In drawing on the theory of autonomous learning, this paper discusses the design framework of stimulating and sustaining learning in educational games, and establishes a bridge between user learning behavior and entertainment behavior. This will provide a theoretical and case study reference for the integration of educational purpose and game entertainment into educational games.

## Introduction

Educational games originated from the concept of “serious game” put forward by Abt (1970) in 1970. He maintained these games are “not primarily about entertainment but about serious, fun content, in which players learn information, gain new learning experiences, and inspire learning motivation and creativity” (Abt, 1970, p. 8). Gee (2007, p. 10) argued: “Good games are problem-solving spaces that create deep learning—learning that is better than what we often see in schools.” A great deal of empirical research confirms that these games play a very positive role in stimulating students’ learning motivation, improving students’ innovation ability, and prompting students to form a good emotional attitude (this is one of the important research directions of educational technology discipline) (Mayer, 2019; Calvo-Morata et al., 2020). The pedagogical potential of games was recognized when the game “America’s Army” was created in 2002. As the research on educational games has continued to grow, the focus of research has gradually shifted from design development and application evaluation to the integrated exploration of education and entertainment. Connolly et al. (2012) has for example highlighted the impact of educational games on learners’ perceptions, behavior, emotion, and motivation, and has particularly emphasized their contribution to knowledge acquisition and content comprehension. Boyle et al. (2016) compares educational games to casual games and finds that educational games contribute significantly to knowledge learning, while casual games mainly produce behavioral, cognitive, emotional, and physiological changes. However, Girard et al. (2013) undertakes practical applied research to assess the acceptability and effectiveness of educational games and finds that only a small percentage of games improved learning, and that most educational games are comparable to traditional teaching methods in terms of knowledge and skill acquisition.

Motivation, as an important factor that motivates users’ behavior to achieve the ultimate goal, permeates the knowledge learning and leisure entertainment aspect of educational games. Intriguing content, such as action scenes, background music, mechanics and good storylines, is naturally entertaining and can motivate users’ entertainment motivation and increase game engagement. But the rich entertainment content of the game can also act to the detriment of learning motivation. This paper discusses the dynamic integration of educational purpose and educational game entertainment from an independent learning angle, and focuses on the improvement and maintenance of learning motivation. In doing so, it constructs the strategy and framework of educational game design.

## Literature review

### Research status

The main purpose of educational games is to learn knowledge and information, and interest games are a medium of knowledge transfer. The integration of education and entertainment in educational games is not a recent problem. Since 2015, researchers have gradually increased their exploration of education and play in educational games. Arnab et al. (2015) has, in integrating educational and recreational elements, proposed the Learning Games (LM-GM) Model. Based on self-explanation theory, Clark et al. (2016) draws on Self-Explanation Theory to propose game design principles such as non-open-ended answers, along with prompts that take the game’s processing needs into account and also mobilize learner participation. Silva (2020) separates the learning content from the entertainment elements and proposes the main steps to define the learning mechanism of educational games. Some scholars have also conducted a balanced study of education and recreation for motivators. Tuzun (2004) constructs a multi-motivation framework for educational games, summarizes 12 types of educational game motivation, and discusses the relationship between elements by drawing on the concept of duality. Tuzun divides the framework into four main areas (subject, activity, outcome, and object), which each consist of two main motivators that interact. Also, Tuzun (2004) highlights the importance of choice in motivating learners, and proposes creativity, learners’ identity expression, and social relationships formed during the playing of online games can motivate learners. Carvalho et al. (2015) proposes the model of serious games (ATMSG) by drawing on Activity Theory. He suggests play and learning coexist in the same player and subject who has different motivations. Academic research on educational game motivation currently mainly focuses on the generalization of factors, but research into its application strategy in educational games is still being explored, and there are few practical cases.

### Self-regulated learning

Self-Regulated Learning (SRL) is a theory which holds that learners are able to proactively plan their learning content, implement learning strategies and constantly monitor, reflect on and adjust their learning behaviors during the learning process (Panadero, 2017). It is essentially a “feedback loop” of sorts, that can be roughly divided into the planning, implementation and reflection stages (Zeidner and Stoeger, 2019; Guo et al., 2021). The planning stage is the self-regulating behavior before learning begins; the implementation stage is the self-regulating behavior during learning; and the reflection stage is the adjustment of the next learning strategy or goal after learning is over—it involves comparing feedback from the objective against feedback from the implementation stage.

### ARCS learning motivation model

Promoting and improving learning motivation has an important role to play in education. Educational games can strongly attract learners and motivate their learning behavior. Malouf (1988) comparison experiment finds that educational games can be effective in maintaining learners’ learning motivation. Chile and Rosas et al. (2003) undertake an empirical study and find that the application of educational games in the classroom can effectively motivate learners and positively affect classroom instruction. Research of motivators in educational games still lacks a consensus, and Malone and Lepper’s study is the most representative of the theory (Laine and Lindberg, 2020). Malone and Lepper put forward a complete theory of individual “intrinsic motivation” and experimentally validate and summarize it. They present challenge, control, curiosity and fantasy as individual factors that increase motivation; and competition, cooperation and respect as collective factors that have the same effect. The biggest difference between video games and learners themselves is that the latter are able to exercise their subjective initiative, make independent choices and regulate their behavior (Winne, 2017). In considering the integration of self-regulation learning theory and educational games, it is crucial to select the appropriate regulatory elements as this will help to maintain and enhance users’ learning motivation through element regulation.

The ARCS model of learning motivation (ARCS) was introduced to filter and refine the elements of regulation in educational games. It categorized factors that influence students’ motivation, including attention – motivation first stems from the fact that the current learning arouses curiosity and attention in the motivated subject; relevance—motivated subjects are more likely to maintain motivation when they feel that the current learning is highly relevant to their own values and experiences; confidence—motivated subjects are more likely to focus on current learning when they believe that they are capable of performing a particular learning task; and satisfaction—motivated individuals are more likely to have a high level of satisfaction and to maintain their motivation in new learning situations when they are fairly evaluated on the completion of a task and have the opportunity to use the content to solve a practical problem (Keller, 1987). ARCS focuses on motivation and on its maintenance. It does not only analyze the mechanism of motivation generation but also tries to identify how to promote motivation generation through external design, in the expectation this will relieve learner anxiety and improve learning initiative (Turel and Ozer Sanal, 2018). ARCS is also often used to assess the effectiveness of educational games (Guo et al., 2021; Laurens-Arredondo, 2022).

## Methodology

In taking learning motivation as its starting point, this paper discusses the balance between entertainment and learning in educational games by using qualitative research methods that include documentary analysis and it also applies inductive methods that include contrast and analysis. This paper proposes a dynamic adjustment strategy based on SRL and ARCS, which can continuously motivate and sustain the user’s learning motivation. The educational game’s design framework is then constructed, and the design practice of the evolutionary educational game of marine and terrestrial creatures is completed by referring to this strategy and framework.

### Game strategy design based on self-regulated learning

**FIGURE 1:**
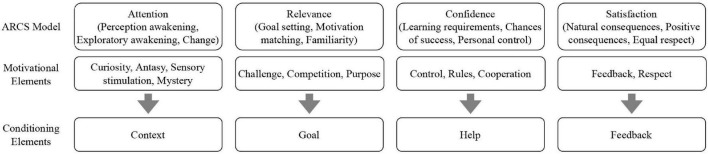
Design of reconciliation elements.

In referring to the design of game strategy, this paper summarizes and analyzes the motivational factors of the ARCS educational game (see Figure 1). A review of the literature shows that while there is no academic consensus on the factors that influence motivation for educational play, some similar factors are generally put forward, including challenges, curiosity, feedback, rules and sensory stimulation. Curiosity, fantasy, mysteries and sensory stimulation that capture the learner’s attention are classified as elements of the game scenario because they are primarily acquired through auditory and visual stimuli related to the game. Challenges, competition and purpose are related to the game’s core mechanism; since the core mechanism of the game serves the game itself, the core mechanism of the educational game introduced by the ARCS is closely related to the game’s pedagogical objectives. Process elements such as control and cooperation, which are associated with students’ learning experiences and are important stages in the development of learners’ self-confidence—they also regulate the difficulty of the game and are accordingly classified as “helpful.” Feedback element is an explicit expression of satisfaction in the ARCS, and feedback directly influences the learners’ satisfaction and sense of achievement. The final paper identified four regulatory elements based on the ARCS: *context*—awakening users’ curiosity and attention; *goal*—managing learning content and objectives; *help*—providing appropriate guidance; and *feedback*—learning behavioral statistics and drawing on related feedback. Context and feedback focus more on auditory, interactive, visual and other elements of entertainment, and targeting and helping emphasize the purposefulness of educational games.

**FIGURE 2:**
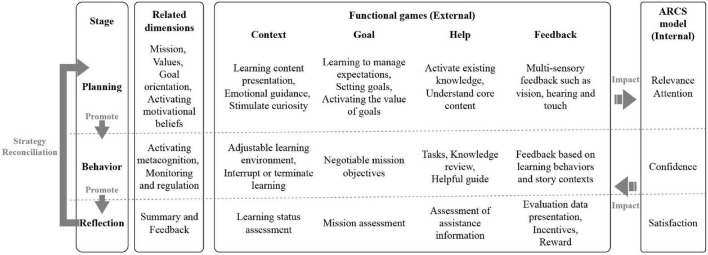
Design of educational game strategies based on self-regulated learning.

The self-regulating process of learning motivation divides game strategy design into three dynamic cyclic stages, specifically the planning, behavior/will control and reflection stages. The four key elements (context, goals, help and feedback) are dynamically tuned to the dimension content to be implemented at each stage (see Figure 2). The planning stage is the beginning of learning behavior; curiosity and challenge drive users to the behavior stage; users’ learning behavior is the content basis of the reflection stage; and the reflection stage provides a data basis for the context, goal, help and feedback regulation in the next cycle.

#### Contextual adjustment

We seek to create a comfortable learning environment for users by adjusting elements such as pictures and audio to their learning content, learning habits and cognitive level. At the planning stage, we need to present the learning content in visual and auditory form and create a learning environment for the user. Through appropriate emotional guidance and sensory stimulation, we arouse users’ curiosity and stimulate their desire to learn. The behavioral phase emphasizes the user’s independent mastery. Users can adjust the game scenario to their own learning schedule and needs. Situational feedback and evaluation record user behavior such as environmental adjustment and learning interruption, and generate corresponding learning status evaluation data.

#### Goal adjustment

In accordance with the user’s learning ability, cognitive level, learning status and other evaluation data, task objectives are dynamically adjusted. The aim is to keep the user in a state of mental flow to the greatest extent possible. In the planning stage, we set appropriate mission objectives for users based on mission evaluation, situation evaluation and ancillary evaluation data from the previous cycle, which enables us to build learning expectations and define learning tasks. In the action phase, users can control their learning behavior, adjust the task goals autonomously and avoid mismatching the task content, and this can cause the game to get out of control. In the reflection phase, study behavior such as duration, task difficulty and operation trajectory are summarized and evaluated in order to provide data support to the next cycle. In this phase, knowledge will also be reviewed and collated to consolidate learning outcomes.

#### Help adjustment

In accordance with users’ cognitive level, learning ability, learning process, and other evaluation data, we adjust in-game operation hints, content review, task guidance and other learning aids. At the planning stage, we adjust the manner and extent of activation of existing knowledge or core content by drawing on user assessment data from the previous cycle. In the behavior phase, the content and frequency of learning aids are adjusted in accordance with the user’s learning behavior and state at this stage. Information on learning assistance of this kind is recorded and evaluated during the reflection phase.

#### Feedback adjustment

Adapt auditory, tactile and visual elements of educational games to the user’s learning behavior, learning stage, and cognitive level, and give psychological and sensory feedback.

The four-game conditioning elements extracted from the ARCS can motivate users from the outside, form internal and external linkages with internal factors (such as attention, relevance, self-confidence and satisfaction), enhance learning willingness and keep educational games alive.

### Game design principles based on self-regulated learning

**FIGURE 3:**
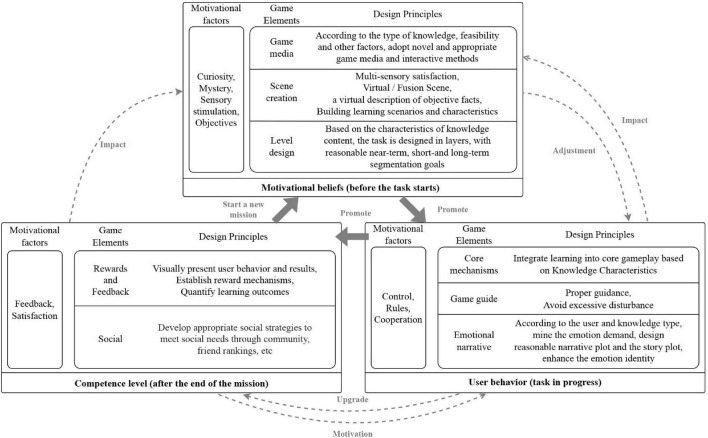
A framework for motivation-oriented educational game design.

The motivation-oriented learning process is summarized in three stages: before, during and after the task (see Figure 3). Sensory stimulation is provided to the user through elements that include game media, scene creation, and horizontal playback before the task starts. This will enhance the user’s curiosity and motivation beliefs. In the mission phase, we strengthen rules and user controls to promote learning behaviors through the core game mechanism, game guidance and emotional storytelling. At the end of the task, user satisfaction can be improved by rewarding feedback and social systems that visualize learning behaviors and outcomes. Motivation before the task stimulates the users’ learning behavior; learning behavior increases users’ knowledge reserve and improves users’ learning ability; and learning ability and pleasant learning experience further strengthens users’ motivation beliefs. These three stages interact to form a dynamic cycle. In drawing on the strategy foundation, this paper puts forward the specific design principles of game elements.

#### Game medium

Performance in games depends on interactive media, and is influenced by aesthetics, dynamics and mechanisms. Educational game media choices should be based on educational themes, technical feasibility and user acceptance and should, to the greatest extent possible, be attractive, innovative and draw on participatory game media or methods. New and avant-garde interactive media have a natural advantage in stimulating curiosity and motivation to learn.

#### Scene creation

In referring to educational content, user group and game type, this paper takes visualization as its starting point, presents objective things virtually and constructs learning scenes, including audio-visual scenes and user roles.

#### Level design

Motivity-controlled educational game design should consider user characteristics and educational content. Detailed short, medium and long-term goals are formulated in conjunction with a step-by-step mechanism that helps users gradually complete educational content and achieve ultimate educational goals.

#### Core mechanism

Educational game design should be based on the intrinsic mechanisms of educational knowledge, and corresponding game content and learning strategies should be designed according to contextual, descriptive and procedural knowledge characteristics. Knowledge learning is the core purpose of educational games, and the design of the core mechanism of games should be combined with educational content. Establish the connection between knowledge and play, let the user game behavior reflect the learning behavior, and help the user to complete cognitive construction in the game.

#### Game guidance

Game guidance and level design jointly affect the intensity of an educational game task. Level targets provide task content; game guidance determines task scaffolding. In the game guidance design, we should develop different guidance modes in accordance with different mission objectives as this will meet the differentiated needs of different types of users. Game coaching should be based on immersive principles as this will help to avoid excessive distraction (Chee and Wong, 2017).

#### Emotional narrative

Educational games are synchronized in narrative space and time. Narrative and emotional articulation around learning content that draw on dialogue, interaction, graphics, music, sound and text provide the basic principles of educational games.

#### Reward and feedback

Visualize the user’s behavior trajectory, learning outcomes, learning status, and ability level, and provide timely feedback on user behavior and actions by drawing on factors such as grades, rewards and scores. Quantitative demonstration of learning effectiveness can help users build self-awareness and improve their satisfaction and sense of achievement.

#### Social

People’s social needs are reflected in cooperation and competition, trading, making friends, socialization, ranking and other games. Games, educational knowledge and user types have different social needs. We should focus on educational goals, complement them with entertainment elements, choose appropriate social functions and avoid excessive or meaningless social content.

#### Educational game design practice

The pandemic, which broke out in late 2019, reaffirmed the importance of ecological civilization in an extreme way. It has accordingly led to an improvement in public ecological civilization behavior and awareness. Surveys show that the implementation of popular ecological civilization science directly affects the public’s sense of meaning, cognitive attitudes and willingness to participate (Nazir et al., 2021). But science popularization activities are often constrained by public awareness, science popularization resources, scientific space and space and talents, and are difficult to carry out continuously on a large scale. The digital education game is simple, convenient, and quick to spread, which is unaffected by time space and material resources and is also an ideal medium for carrying out science popularization activities (Mattioli, 2021). This paper therefore selected biological evolution as a learning component of an educational game and designed an educational game (“Ocean to Land”) to assist vertebrate evolution. It seeks to help users understand the evolutionary path of vertebrates, the general laws of species evolution (including the internal and external factors that affect species evolution), deepen users’ understanding and knowledge of life, expand users’ knowledge base of life sciences and improve ecological literacy.

#### Game overview

**FIGURE 4:**
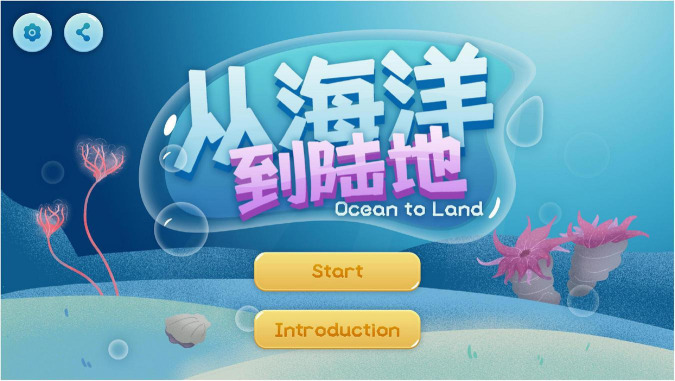
“Ocean to Land” game home page.

The game takes key nodes of vertebrate fish landing evolution as its levels, divided by evolutionary time into vertebrate origin, jawed fish, bony fish, carnivorous fin fish, and tetrapods. The game incorporates the general laws of biological evolution into the core mechanics game, and shows users a Paleozoic marine landscape that reveals interesting aspects of species’ evolution mode, context and survival environment (see Figure 4). The game was currently completed as a lightweight web application, mainly using HTML5 and JavaScript. It is a cross-platform game that can run on various devices, such as tablets, computers, and cell phones.

#### Core mechanism design

**FIGURE 5:**
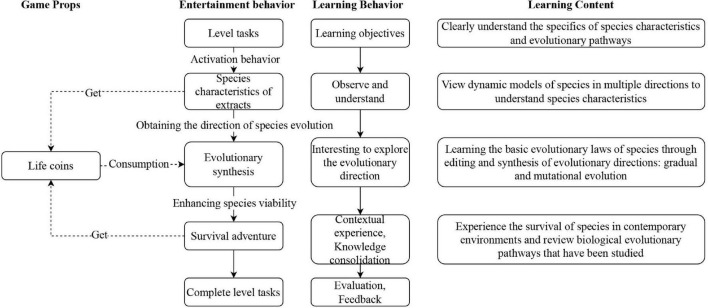
Core game mechanics.

In drawing on the game design principle of self-regulated learning, the game combines biological “principles of balanced intermittent evolution” with the core game mechanism, and this creates mirrored relationships between entertainment and learning behaviors that stimulate learning motivation and behavior (see Figure 5). An understanding of speciation and the direction of evolution are central to the game. Players can achieve their goals through three main gameplay methods, specifically species feature extraction, evolutionary synthesis and survival adventure. In the game, players can observe animals, explore evolutionary directions, experience survival and learn about vertebrate evolutionary pathways.

In “Species Extraction,” players observe the fish’s dynamics, learn about its features and gain an insight into its evolutionary direction and life coin. In the “Survival Adventure” module, players manipulate the species to capture prey in the ocean and evade predators, who are converted into life coins. The “Evolution Synthesis” module asks players to edit or synthesize evolutionary directions on the basis of mission descriptions and prompts, and enables them to explore and decipher species evolution and mutations. Editing or synthesis operating this part requires consuming the life coin, and the consequences of evolution can affect the species’ ability to survive in “survival adventures.”

#### Game regulation strategy

The game is based on the strategic framework of SRL, which influenced the refinement design of the game context and mission goals by providing helpful guidance and feedback. Table 1 decomposes the game functions on the basis of the characteristics of vertebrate fish evolution science, and proposes three functional partitions (simplified, basic, and extended). The functions that are more fun but that place more demands on players’ learning ability are divided into the expansion partition. The functions that make it easier and faster to obtain information but which are not entertaining are divided into the simplified partition. The game uses basic features and then adjusts them on the basis of the players’ game evaluation data.

**TABLE 1:** Game function division.

	Game context	Mission goals	Helpful guidance	Feedback
Simplified	Voice guidance; message reading; dialogue pop-up boxes	Complete decomposition of core tasks into sub-tasks	Demonstrating acquired knowledge; step-by-step instructions when there is no operation for a long time	Voice encouragement
Basic	Key text messages; interactive animation	Break down key points of core tasks into sub-tasks	Hide mastered knowledge; auto-tips in the form of cards according to the game progress	Ambient music; sound effects; exercise effect
Extended	Animal behavior guidance; hidden messages	Core missions	Hide mastered knowledge; no active prompting, users review on their own	Enhances the user’s tactile senses with vibrations when triggering mutational evolution

### Context

**FIGURE 6:**
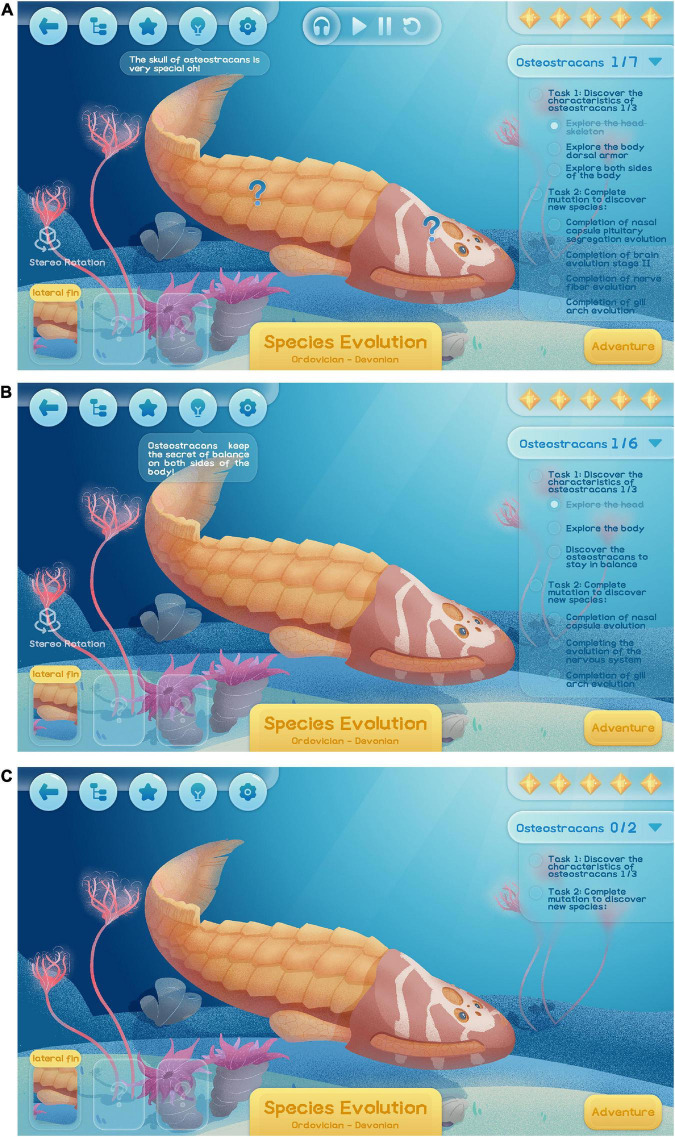
Species feature extraction page.

In referring to the characteristics and evolutionary knowledge of fish, the game is based on the ocean of related periods, and it seeks to construct an adjustable learning environment. For example, in Figure 6B, which is the basic situation of “feature extraction,” the game takes the ocean scene, fish model and operation method reminder as its basic functions. When the player has a high exploration ability, text messages and operational prompts are reduced to reduce information disturbance (see Figure 6C). When a player’s information acquisition ability and cognitive level do not meet the requirements of basic functions, voice messages are added to help the player understand the game (see Figure 6A).

### Goals

In accordance with the characteristics of scientific and technological knowledge, the game’s mission objectives are mainly observation tasks and exploration tasks. Take the bone armor fish level as an example. As Figure 6B shows, the basic function is the core exploration task to “discover the characteristics of bone armor fish”—this refers to the disassembled head, back and balance, and involves three subdivision tasks. The core exploration to discover the initial full-jawed fish involves three sub-tasks that relate to the nasal sac, nervous system and gill arch. Core tasks are only retained when players have a high ability to learn and explore (see Figure 6C). When players are less able to learn and explore, tasks are further broken down (see Figure 6A).

### Help

The in-game help function mainly consists of information tips and instructions. As Figure 6B shows, the basic function is to trigger an information cue in the upper left corner if the player has not explored the action correctly for a long time. As players learn more, in-game activity alerts will be canceled and players can choose to view them independently (see Figure 6C). If players have limited learning abilities, detailed instructions are provided (see the hint in Figure 6A about the characteristic location of oracle fish).

### Feedback

The basic purpose of game feedback is to provide audio and visual feedback through background music, sound effects and kinetic effects. The expansion strategy seeks to add vibration feedback at key points in survival adventure, including by completing core roles and adding cell phone gravity feedback. Voice encouragement can be added when players have limited learning abilities and are impatient.

## Discussion and conclusion

The article constructs a framework of strategies and design principles based on self-regulated learning, and is guided by strategies and principles applied in the design and development of the educational game “Ocean to Land.” This game explores the evolutionary characteristics of vertebrates through fish models, and allows players to grasp the general law of animal evolution and the “balancing discontinuity principle.” It also enables players to consolidate their knowledge and fully explore evolutionary directions through survival adventures. In drawing on the regulatory strategy, Marine International has designed a functional regulation that consists of four elements, specifically context, goals, help and feedback. In drawing on statistics related to player learning interruptions, we refer to operation intervals, level duration, frequency of help, point of life consumption and number of failed attempts to survive, and formulate specific rules and measures of segmentation that have practical application implications for exploring the balance of entertainment and teaching in educational games. When compared with educational games with relatively fixed task challenges and game scenarios (e.g., “U.S. Army”), the task settings and learning scenarios are more flexible and can be more easily adapted to the learning needs of different learner types. Subsequent studies, in considering the effects of different game types and learning populations on the learning effectiveness of educational games, will provide a more detailed assessment of the effectiveness of the “Ocean to Land” framework by undertaking user experiments.

Learning knowledge and skills is the fundamental purpose of educational games, and video games provide ways and means of disseminating educational content. This paper emphasizes the learning purposefulness of educational games and explores the integration of education and entertainment in educational games from the perspective of learning motivation. On the basis of the SRL, the four main regulation elements of context, goal, help, and feedback are extracted from the combination of motivation elements in educational games and integrated with the ARCS. The dynamic control strategy of the educational game is then constructed, and the design principle of the educational game is put forward in accordance with the three stages of intermediary control. In being guided by regulatory strategies and design principles, and operating in the pandemic’s wave of eco-civility, “Ocean to Land,” an educational game on biological evolution, was introduced and developed. This paper seeks to explore the dynamic integration of educational purposes into educational game entertainment forms; in drawing on motivating factors to construct a mapping bridge between users’ learning behavior and entertainment behavior, this paper provides points of theoretical and practical reference that will influence the future research and development of educational games.

## Data availability statement

The original contributions presented in this study are included in the article/supplementary material, further inquiries can be directed to the corresponding author/s.

## Author contributions

FJ and DS made the theoretical design of this article, reviewed, and revised the manuscript. FJ drafted the manuscript. All authors have read and reviewed the final manuscript.
